# Factors influencing the production of stilbenes by the knotweed, *Reynoutria *× *bohemica*

**DOI:** 10.1186/1471-2229-10-19

**Published:** 2010-01-29

**Authors:** Marcela Kovářová, Kristýna Bartůňková, Tomáš Frantík, Helena Koblihová, Kateřina Prchalová, Miroslav Vosátka

**Affiliations:** 1 Institute of Botany, Czech Academy of Science, Průhonice 1, 252 43, Czech Republic; 2 Research Institute of Organic Syntheses, Rybitví 296, 533 54 Pardubice, Czech Republic

## Abstract

**Background:**

Japanese knotweed, *Reynoutria japonica*, is known for its high growth rate, even on adverse substrates, and for containing organic substances that are beneficial to human health. Its hybrid, *Reynoutria *× *bohemica*, was described in the Czech Republic in 1983 and has been widespread ever since. We examined whether *Reynoutria *× *bohemica *as a medicinal plant providing stilbenes and emodin, can be cultivated in spoil bank substrates and hence in the coalmine spoil banks changed into arable fields. We designed a pot experiment and a field experiment to assess the effects of various factors on the growth efficiency of *Reynoutria *× *bohemica *on clayish substrates and on the production of stilbenes and emodin in this plant.

**Results:**

In the pot experiment, plants were grown on different substrates that varied in organic matter and nutrient content, namely the content of nitrogen and phosphorus. Nitrogen was also introduced into the substrates by melilot, a leguminous plant with nitrogen-fixing rhizobia. Melilot served as a donor of mycorrhizal fungi to knotweed, which did not form any mycorrhiza when grown alone. As expected, the production of knotweed biomass was highest on high-nutrient substrates, namely compost. However, the concentration of the organic constituents studied was higher in plants grown on clayish low-nutrient substrates in the presence of melilot. The content of resveratrol including that of its derivatives, resveratrolosid, piceatannol, piceid and astringin, was significantly higher in the presence of melilot on clay, loess and clayCS. Nitrogen supplied to knotweed by melilot was correlated with the ratio of resveratrol to resveratrol glucosides, indicating that knotweed bestowed some of its glucose production upon covering part of the energy demanded for nitrogen fixation by melilot's rhizobia, and that there is an exchange of organic substances between these two plant species. The three-year field experiment confirmed the ability of *Reynoutria *× *bohemica *to grow on vast coalmine spoil banks. The production of this species reached 2.6 t of dry mass per hectare.

**Conclusions:**

Relationships between nitrogen, phosphorus, emodin, and belowground knotweed biomass belong to the most interesting results of this study. Compared with melilot absence, its presence increased the number of significant relationships by introducing those of resveratrol and its derivatives, and phosphorus and nitrogen. Knotweed phosphorus was predominantly taken up from the substrate and was negatively correlated with the content of resveratrol and resveratrol derivatives, while knotweed nitrogen was mainly supplied by melilot rhizobia and was positively correlated with the content of resveratrol and resveratrol derivatives.

## Background

Invasive, even transformer, species [1-3] of the genus *Reynoutria *are plants that have many potential applications due to their high genotypic variability, their high growth potential and the quality of their biomass. Because they efficiently cover waste substrates even under adverse environmental conditions, these species may be useful for revitalizing man-made landscape features such as ash deposits or coalmine spoil banks. Restrictions must be set in place to prevent the spread of these plants into the surrounding landscapes. Our aim was to test the efficiency with which the production of resveratrol, resveratrol derivatives and emodin could be stimulated in *Reynoutria *× *bohemica*, as well as to evaluate the suitability of clayish coalmine spoil banks for pharmaceutical production. These substrates do not contain heavy metals and there is no danger of the spread of knotweed by water because coalmine spoil banks are far from running water bodies. There are waste areas composed of these substrates waiting for reclamation and revegetation in the Czech Republic, and the cultivation of knotweed for pharmaceutical use would require only a few acres of land in order to meet the market demands. To our knowledge, there have been no attempts to date to grow knotweed, namely *R. *× *bohemica*, for pharmaceutical use as a medicinal plant.

The spoil banks examined in this study were formed by clay deposited during the removal of materials overlaying brown coal, which has been mined extensively from large areas in northern and western Bohemia. Reclamation of these nitrogen-deficient clay deposits requires long periods of time; therefore, processes that promote the revegetation of these areas are of great interest. Thus, we planted knotweed in an experimental arable field near coal mines that was composed of clay deposits, and aimed to track the growth rates and the production of stilbene and emodin under field conditions over a three year period. Clay was also used as a substrate in our two-year pot experiment in combination with other reclamational substrates such as loess, compost and a slow-soluble natural fertilizer.

*Reynoutria *× *bohemica *[4] has been described in the Czech Republic as a hybrid species of *R. japonica *Houtt. var. *japonica *and *R. sachalinensis *(F. Schmidt) Nakai. This species has become widespread due to its high genetic diversity, eco-plasticity, and growth rate. Because *R. japonica *is well known and has been used for stilbene production, we sought to determine whether the hybrid species could be used for a similar purpose.

The main aim of this study was to test the suitability of different substrates for knotweed growth and for the production of resveratrol, its derivatives, and emodin. Resveratrol (3,4',5-trihydroxystilbene; molecular weight 228.2 g/mol) is a naturally occurring plant polyphenol that is present in grapes, berries, and peanuts in significant levels. It has been shown to have antifungal [5], antioxidant, antimutagenic, anti-inflammatory, chemopreventive [6,7], and cytotoxic effects in different tumour cell lines [8-11] including those of breast cancer [12]. Knotweed is a plant that is traditionally used for the production of resveratrol in Asia, and particularly in China. In Europe, wine is the main source of this substance; a variety of stilbenes have been found in wine, including astringin [13], cis- and trans-piceid, trans-resveratrol and astringin [14], trans-astringin, trans-piceid, trans-resveratrol and cis-resveratrol [15,16], trans-astringin, cis- and trans-piceid, and cis- and trans-resveratrol. In addition to studying the potential of "inland" sources of resveratrol in *R. *× *bohemica*, we also wanted to determine the content of other stilbenes in this plant and to assess the contributions of its different components to the production of these compounds. It has been suggested that resveratrol-glucosides (e.g., piceid) are degraded in the gut by bacteria and that resveratrol is then released [17-19], thereby increasing the amounts of resveratrol available to the organism. Measuring all of the stilbenes present is thus important, so we monitored the full range of resveratrol-containing substances, apart from emodin.

Under harsh conditions, plants would be expected to possess advantageous features, such as mycorrhizal symbiosis, that would enable them to overcome the challenges of their environment. *Melilotus *(both *M. albus *Desr. and *M. officinalis *(L.) Lam) is a typical plant that is capable of surviving, and even thriving, on low-nitrogen spoil banks due to the presence of mycorrhiza and nitrogen-fixing rhizobia [20,21]. Both the parental species of *Reynoutria *× *bohemica *are, however, described as non-mycorrhizal species [22]. The hybrid is therefore also expected to be non-mycorrhizal. Surprisingly, mycorrhizal colonisation was found in the roots of *R. *× *bohemica *sampled from an *Alnus glutinosa *forest (J. Rydlová, personal communication). An arbuscular type of mycorrhiza was also found in the roots of knotweed plants growing on the volcanic soils of Mt. Fuji, Japan [23]. We therefore wanted to determine whether the experimental introduction of mycorrhizal fungi to knotweed roots with a nurse plant [24,25] might stimulate the production of resveratrol and its derivatives.

We designed a pot experiment in which *R. *× *bohemica *was grown on different substrates with or without *Melilotus alba *(white melilot), a plant typically occupying spoil banks. We hypothesized that melilot could serve as a potential donor of mycorrhizal fungi and would also increase soil nitrogen content.

## Results

### Pot Experiment

Table 1 provides an overview of the results of the **pot experiment**.

**Table 1:** Overview of plant characteristics tested using an ANOVA during the two years of the pot experiment

Plant characteristics measured		Significance of factors and their interactions						
		**A**	**B**	**C**	A*B	A*C	B*C	A*B *C
		**year**	**substrate**	**melilot**				

Plant aboveground characteristics	in year							

Knotweed								

Branch no	2006+07	0.001	0.001	NS	0.001	NS	NS	NS

Plant dry mass (g)	2006+07	0.001	0.001	0.001	NS	0.01	NS	NS

Leaf area (cm^2^)	2007	x	0.001	NS	x	x	NS	x

Melilot								

Plant dry mass (g)	2006+07	0.05	NS	x	NS	x	x	x

								

Plant belowground characteristics								

Knotweed								

Root and rhizome dry mass (g)	2007	x	0.001	0.001	x	x	NS	x

Root colonisation rate F (%)	2006+07	0.001	0.001	x	0.05	x	x	x

Root colonisation rate M (%)	2006+07	0.001	0.001	x	NS	x	x	x

Nitrogen (%)	2006+07	0.001	0.001	0.001	0.001	0.001	0.001	0.001

Carbon (%)	2006+07	NS	NS	NS	NS	NS	NS	NS

Phosphorus (ppm)	2006+07	0.001	0.001	0.001	NS	NS	NS	NS

Astringin (mass %)	2006+07	0.001	0.01	NS	NS	0.01	0.01	NS

Astringin 2 (mass %)	2006+07	0.001	0.05	NS	NS	0.05	0.01	NS

Piceatannol (mass %)	2006+07	0.01	0.001	0.05	0.001	NS	NS	NS

Piceid (mass %)	2006+07	0.001	0.05	0.01	NS	NS	NS	NS

Resveratrol (mass %)	2006+07	NS	0.001	0.001	0.05	NS	0.05	NS

Resveratrolosid (mass %)	2006+07	0.001	NS	0.01	NS	NS	0.01	NS

Emodin (mass %)	2006+07	0.001	0.001	0.001	NS	0.001	NS	NS

Resveratrol-derivatives (mass %)	2006+07	0.01	0.01	0.001	NS	NS	0.001	NS

Melilot								

Melilot colonisation rate F (%)	2007	x	NS	x	x	x	x	x

Melilot colonisation rate M (%)	2007	x	NS	x	x	x	x	x

x = non-tested

NS = non-significant

**Figure 1:**
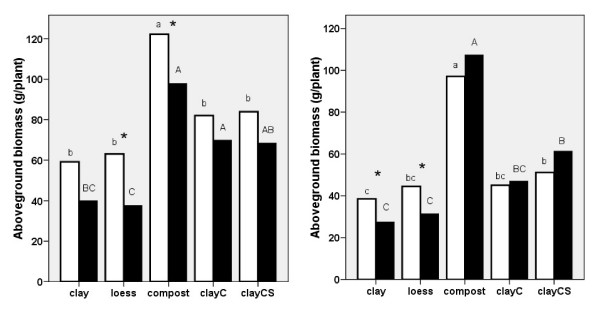
**Aboveground biomass (d.w.) of *Reynoutria *× *bohemica *grown in pots with various substrates based on miocene clay from coalmine spoil banks with (black columns) and without (open columns) *Melilotus alba *(significant differences are indicated by asterisks) in 2006 (a - left) and 2007 (b - right)**. ClayC = clay enriched with slow-release biofertilizer Conavit; ClayCS = clay enriched with Conavit and arbuscular-mycorrhizal product Symbivit, both produced by Symbiom Ltd. Equal letters indicate non-significant differences between the substrates; lower case - without melilot, upper case - with melilot.

*The aboveground biomass *of knotweed showed several significant differences between the substrates in 2006 and 2007 (Fig. 1). The highest biomass was produced in plants grown on compost in both years. There was also a difference observed between plants grown on clay and clayCS in 2007. Similar results were obtained for knotweed grown with melilot. The growth of melilot was unrestricted in 2006, which resulted in competition between melilot and knotweed. The presence of melilot significantly decreased the biomass of knotweed grown on loess and compost. However, decreasing knotweed biomass was noted in all of the substrates (Fig. 1a). A significant decrease of knotweed biomass in the presence of melilot was also noted in 2007 when melilot growth was restricted, but this was only observed for the two low-nutrient substrates, clay and loess (Fig. 1b).

There was a significant difference in the *lateral branch number *of knotweed plants between 2006 and 2007. Relatively high numbers of lateral branches (7-20) were found in 2006, and these numbers decreased significantly in 2007 to 9 and 5 in plants grown on compost in the presence and absence of melilot, respectively. The numbers of lateral branches were reduced further to 0-2 in plants grown on the other substrates (data not shown).

**Figure 2:**
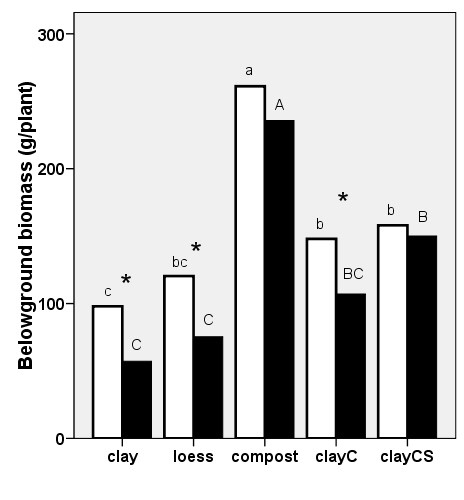
**Belowground biomass (d.w.) of *Reynoutria *× *bohemica *grown in pots with various substrates based on miocene clay from coalmine spoil banks with (black columns) and without (open columns) *Melilotus alba *(significant differences are indicated by asterisks) in 2007**. ClayC = clay enriched with slow-release biofertilizer Conavit; ClayCS = clay enriched with Conavit and arbuscular-mycorrhizal product Symbivit, both produced by Symbiom Ltd. Equal letters indicate non-significant differences between the substrates; lower case - without melilot, upper case - with melilot.

The *belowground biomass *of knotweed was only measured in 2007. Belowground biomass was significantly lower in plants grown on clay, significantly higher in plants grown on clay enriched with nutrients, and was highest in plants grown on compost. The belowground biomass of plants grown on loess was intermediate between plants grown on clay and those grown on enriched clay. The presence of melilot decreased the underground biomass of knotweed grown on clay, clayC, and loess (Fig. 2).

**Figure 3:**
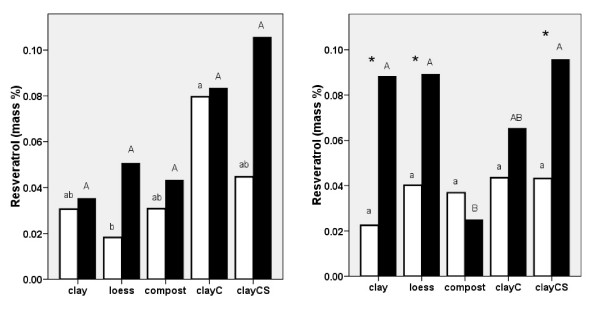
**Resveratrol content in *Reynoutria *× *bohemica *roots and rhizomes grown in pots with various substrates based on miocene clay from coalmine spoil banks with (black columns) and without (open columns) *Melilotus alba *(significant differences are indicated by asterisks) in 2006 (a - left) and 2007 (b - right)**. ClayC = clay enriched with slow-release biofertilizer Conavit; ClayCS = clay enriched with Conavit and arbuscular-mycorrhizal product Symbivit, both produced by Symbiom Ltd. Equal letters indicate non-significant differences between the substrates; lower case - without melilot, upper case - with melilot.

*The *percentage *content of resveratrol *in knotweed rhizomes and roots was higher in the presence of melilot in 2007, except in the case of knotweed grown on compost and clayC. Similar but non-significant trends were observed in 2006. Generally, the highest concentrations of resveratrol were found in plants grown on clayCS in the presence of melilot. The lowest concentrations were found in plants grown on loess without melilot in 2006 (Fig. 3). Piceid is a glucoside of resveratrol. The content of this piceid was also significantly higher in the presence of melilot for plants grown on clay and loess (data not shown). These results suggest that melilot may stimulate the production of glucosides in knotweed grown on low-nutrient substrates.

**Figure 4:**
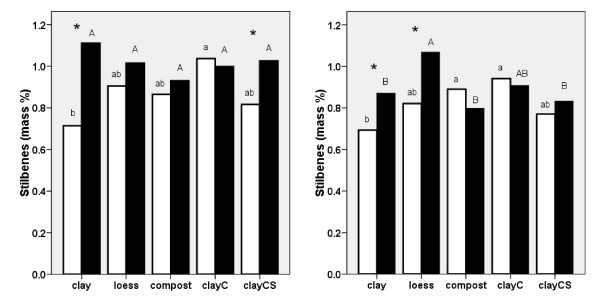
**Resveratrol contained in all its derivatives was measured in *Reynoutria *× *bohemica *roots and rhizomes grown in pots with various substrates based on miocene clay from coalmine spoil banks with (black columns) and without (open columns) *Melilotus alba *(significant differences are indicated by asterisks) in 2006 (a - left) and 2007 (b - right)**. ClayC = clay enriched with slow-release biofertilizer Conavit; ClayCS = clay enriched with Conavit and arbuscular-mycorrhizal product Symbivit, both produced by Symbiom Ltd. Equal letters indicate non-significant differences between the substrates; lower case - without melilot, upper case - with melilot.

Resveratrol and its derivatives, including the glycosidic and aglyconic *stilbenes*, resveratrol, piceatannol, piceid and astringin, were significantly higher in plants grown in the presence of melilot on clay (2006 and 2007), loess (2007) and clayCS (2006; Fig. 4a and 4b). In the absence of melilot, the highest concentration of resveratrol derivatives was found in plants grown on clayC and the lowest was found in plants grown on clay in both 2006 and 2007. In 2006, higher concentrations of resveratrol derivatives were recorded for plants grown in the presence of melilot on loess, but in 2007 the effect of substrate was not significant.

**Figure 5:**
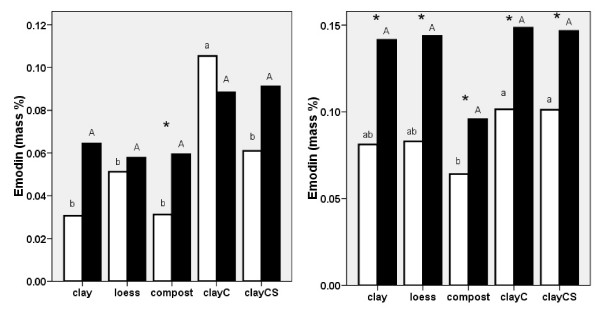
**Emodin content in *Reynoutria *× *bohemica *roots and rhizomes grown in pots with various substrates based on miocene clay from coalmine spoil banks with (black columns) and without (open columns) *Melilotus alba *(significant differences are indicated by asterisks) in 2006 (a - left) and 2007 (b - right)**. ClayC = clay enriched with slow-release biofertilizer Conavit; ClayCS = clay enriched with Conavit and arbuscular-mycorrhizal product Symbivit, both produced by Symbiom Ltd. Equal letters indicate non-significant differences between the substrates; lower case - without melilot, upper case - with melilot.

*Emodin *was significantly higher in plants grown in the presence of melilot on compost in 2006 and in plants grown on all substrates in 2007 (Fig. 5a and 5b). In the absence of melilot, a high concentration of emodin was found in plants grown on clayC in 2006. A low concentration of emodin was found in plants grown on compost in 2007. In the presence of melilot, the effect of substrate was not significant in either year.

**Figure 6:**
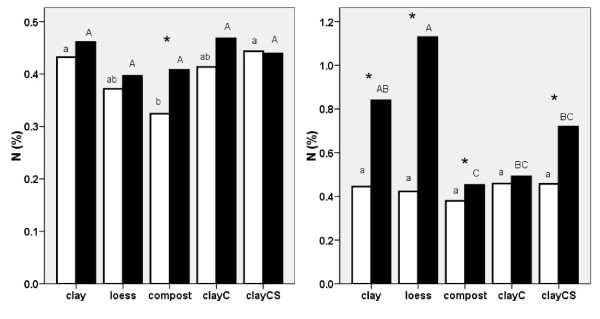
**Nitrogen content in *Reynoutria *× *bohemica *roots and rhizomes grown in pots with various substrates based on miocene clay from coalmine spoil banks with (black columns) and without (open columns) *Melilotus alba *(significant differences are indicated by asterisks) in 2006 (a - left) and 2007 (b - right)**. ClayC = clay enriched with slow-release biofertilizer Conavit; ClayCS = clay enriched with Conavit and arbuscular-mycorrhizal product Symbivit, both produced by Symbiom Ltd. Equal letters indicate non-significant differences between the substrates; lower case - without melilot, upper case - with melilot.

In the presence of melilot, the *nitrogen *concentration of knotweed rhizomes and roots only increased in plants grown on compost in 2006, while in 2007, it increased in plants grown on all substrates except for clayC. Though nitrogen concentrations in knotweed grown without melilot were equal for plants grown on all substrates, nitrogen concentrations were highest in knotweed grown with melilot grown on the two low-nutrient substrates, loess and clay (Fig. 6). The effect of melilot was more pronounced in the second year of the experiment, particularly with respect to plants grown on clay, loess and clayCS. In terms of nitrogen production (g/plant), the highest levels in knotweed roots and rhizomes were found when plants were grown on compost (both with and without melilot) and on clayCS (with melilot). These plants accumulated approximately one gram of nitrogen in their belowground structures, which is about twice as much as that observed in plants grown on clay and/or loess.

*Carbon *concentration in knotweed roots and rhizomes was not affected by the presence of melilot, except in plants grown on loess in 2006 (not shown). There was a positive correlation between carbon and the concentrations of resveratrol derivatives in 2006, both in the absence (r = 0.610***, n = 25) and presence (r = 0.604***, n = 25) of melilot, suggesting that a substantial proportion of organic carbon was bound in resveratrol and its derivatives.

**Figure 7:**
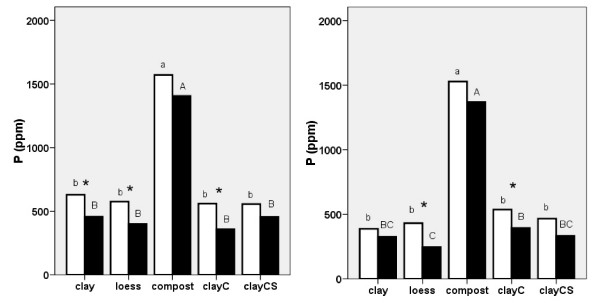
**Phosphorus content in *Reynoutria *× *bohemica *roots and rhizomes grown in pots with various substrates based on miocene clay from coalmine spoil banks with (black columns) and without (open columns) *Melilotus alba *(significant differences are indicated by asterisks) in 2006 (a - left) and 2007 (b - right)**. ClayC = clay enriched with slow-release biofertilizer Conavit; ClayCS = clay enriched with Conavit and arbuscular-mycorrhizal product Symbivit, both produced by Symbiom Ltd. Equal letters indicate non-significant differences between the substrates; lower case - without melilot, upper case - with melilot.

*Phosphorus *in knotweed rhizomes showed similar values in 2006 as in 2007. The concentration of phosphorus in melilot decreased in both years in plants grown on loess and clayC, and in plants grown on clay in 2006. However, there was a distinct trend of reduced phosphorus levels in plants grown on all substrates. The highest concentration of phosphorus was found in knotweed grown on compost with and without melilot in both 2006 and 2007 (Fig 7a, b). The same results were obtained using the production data (phosphorus, g/plant) due to the positive correlation between phosphorus and knotweed biomass.

**Figure 8:**
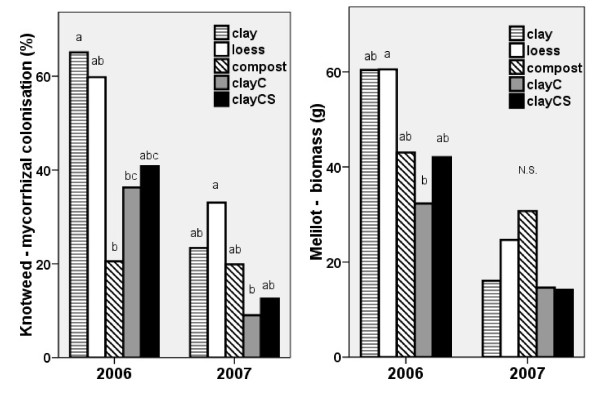
**Mycorrhizal colonization F% of *Reynoutria *× *bohemica *roots grown with melilot (a - left) and aboveground biomass of *Melilotus alba *(b - right), in pots with various substrates based on miocene clay from coalmine spoil banks in 2006 and 2007**. ClayC = clay enriched with slow-release biofertilizer Conavit; ClayCS = clay enriched with Conavit and arbuscular-mycorrhizal product Symbivit, both produced by Symbiom Ltd. Equal letters within the same year indicate non-significant differences between the substrates.

Mycorrhizal colonisation was found only in the roots of knotweed grown with melilot; melilot appeared to serve as a mycorrhiza donor for knotweed. A positive correlation was observed between the mycorrhizal colonisation of knotweed and melilot biomass in both 2006 (r = 0.618***) and 2007 (r = 0.531***), Fig. 8b. The mycorrhizal colonisation rate was higher (20-65%) in 2006, when the growth of melilot was not suppressed, than in 2007 (10-35%). In 2006, the lowest colonisation rate was found in plants grown on compost, while in 2007, plants grown on clay with Conavit had the lowest rate of colonisation (Fig. 8a). In both years, the highest colonisation rate was found in plants grown on nutrient-poor substrates, clay and loess. Although the degree of mycorrhizal infection in melilot did not differ between the substrates (not shown), there was a higher mycorrhizal colonisation of knotweed due to melilot when knotweed was grown on low-nutrient substrates than when knotweed was grown on fertile substrates.

### Field experiment

**Figure 9:**
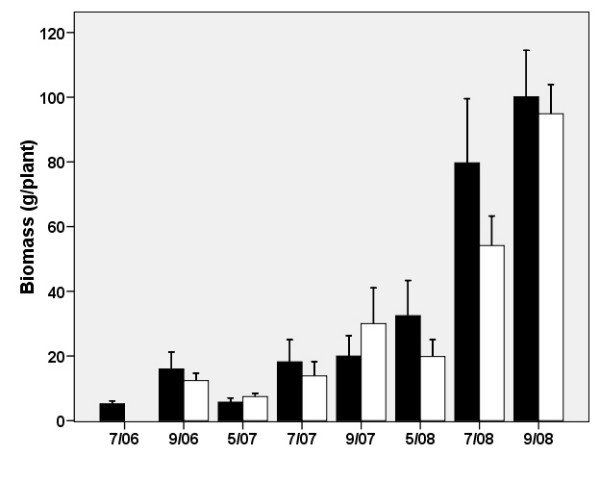
**Aboveground (black columns) and belowground (open columns) biomass (d.w.) of *Reynoutria *× *bohemica *grown in a spoil bank changed into arable field, from April 2006 (planted) to September 2008**. Means ± S.E. indicated.

**Figure 10:**
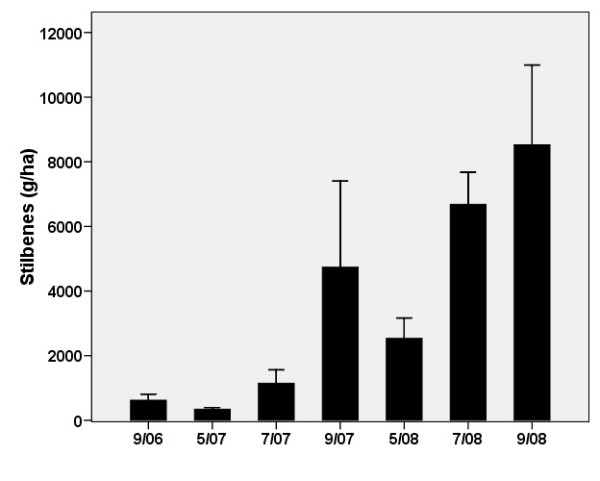
**Stilbenes (resveratrol and resveratrol in its derivatives) in belowground biomass of *R. *× *bohemica *grown in a spoil bank changed into arable field from April 2006 (planted) to September 2008**. Means ± S.E. indicated.

The growth rate and production of stilbene and emodin in the same knotweed clone of *R. *× *bohemica *were examined under field conditions from 2006 to 2008 to investigate the potential for industrial cultivation. Data serving to compare the biomass and production of stilbenes between the field and pot conditions are shown in Figs. 9 and 10, respectively. Substrates in arable fields were most similar to the clay and loess substrates used in the pot experiment, both in terms of particle size and chemical composition. Though the biomass values are comparable, the pot experiment yielded a relatively high belowground biomass in the second year of the experiment (110 g/plant, d.w.), whereas comparable values were not reached by plants grown in the field until the third year (95 g/plant, d.w.). The between-year reduction of knotweed aboveground biomass (from 61 to 42 g/plant, d.w.) observed in the pot experiment due to lateral branch reduction was not observed in the field. In the field, the following values were measured in September 2006, 2007 and 2008, respectively: 16, 20 and 100 g/plant (d.w.).

The content of stilbenes shown in Fig. 10 revealed a high seasonal transfer (translocation) of biomass, as the values of spring belowground biomass (and stilbenes) were lower in both years than those of the preceding autumn. Thus, it is clear that the best time to harvest the belowground biomass of knotweed for stilbenes is the autumn (September). The yield of stilbenes observed at the end of the third growing season (8.5 kg/ha) is promising.

## Discussion

Our three-year basic field experiment enabled us to verify, under field conditions, some of the conclusions of the two-factor pot experiment. The production of both knotweed biomass and stilbenes was comparable in the pots and in the field. The longer period required to attain a substantial level biomass in the field was due to a long period of summer drought at the beginning of the field experiment. The field experiment, in which knotweed production reached 2.6 t dry mass per hectare, confirmed that some of the vast coalmine spoil banks can be used for the targeted production of *Reynoutria *× *bohemica *for pharmaceutical use.

In a well established knotweed stand in Loughborough, UK, [26] reported nearly 16 t/ha of belowground biomass for *R. japonica *in the upper 25 cm of the soil layer. Our expectation is that extensive growing of more productive species of *R*. × *bohemica *on low-fertile soils with no irrigation would produce a biomass of up to 10 t/ha and would contain 80 kg of stilbenes.

In the pot experiment, we observed an interesting interaction between the two main factors, the substrate and the presence of melilot, which affected the production of resveratrol and its derivatives (stilbenes) and emodin. Figs. 4 and 5 show that melilot increased the concentration of resveratrol derivatives and emodin in plants grown on low-nutrient substrates. In general, the effect of melilot appeared to be more pronounced than the effect of the substrates. This was revealed by smoothing the extreme values detected for the levels of resveratrol, its derivatives and those of emodin.

We found that a large amount of biomass was produced on compost with a high concentration of phosphorus and a low concentration of nitrogen (Fig. 6 and 7), giving very low average N:P ratio (2.1 in 2006 and 2.5 in 2007). This suggests that the growth-limiting nutrient in compost is nitrogen, not phosphorus. This is in accordance with the evidence brought by [27] indicating that N limitation might occur when the N:P ratio is as high as 5.8. On the other hand, the nitrogen and phosphorus contents of all of the other (low-organic) substrates were much lower (Tab. 2) and biomass values of knotweed plants grown on these substrates were lower and had lower phosphorus values but similar nitrogen values as the plants grown on compost (the N:P ratio on clay was 7.1 in 2006 and 11.6 in 2007; on loess, ratios were 6.6 in 2006 and 10.0 in 2007). The concentration of nitrogen was substantially higher (twice on clay and even more on loess) in the presence of melilot, while the concentration of phosphorus decreased (the N:P ratio on clay was 10.4 in 2006 and 28.3 in 2007, and on loess ratios were 9.9 in 2006 and 46.6 in 2007). This suggests that on clay and loess, phosphorus limits or co-limits [27,28] the growth of knotweed and that knotweed accumulates nitrogen but not phosphorus. The limitation of phosphorus reported by [29] was due to a N:P ratio greater than 16, while in [30] this effect was due to a N:P ratio greater than 20.

We provide the following explanation for the low nitrogen fixation observed only on compost. Nitrogenase is known to be sensitive to oxygen. Oxygen-free areas within the plant roots are thus created by the binding of oxygen to haemoglobin, which ensures anaerobic conditions necessary for nitrogen fixation http://www.biologie.uni-hamburg.de/b-online/e34/34b.htm.

Compost is a well aerated substrate, especially in contrast to clay or loess. Lower nitrogen fixation is thus expected in compost in comparison to clayish substrates. Indeed, our data from the second year of the pot experiment showed large quantities of nitrogen accumulated by melilot on low-nutrient clay and loess substrates but not on compost (Fig. 6b). This finding agrees well with field observations that melilot grows well on heavy, clayish soils but not on organic substrates.

In contrast to nitrogen, phosphorus was predominantly taken up from soil substrates. Knotweed deposited surplus amounts of phosphorus in rhizomes, especially when plants were grown on high-phosphorus compost.

**Figure 11:**
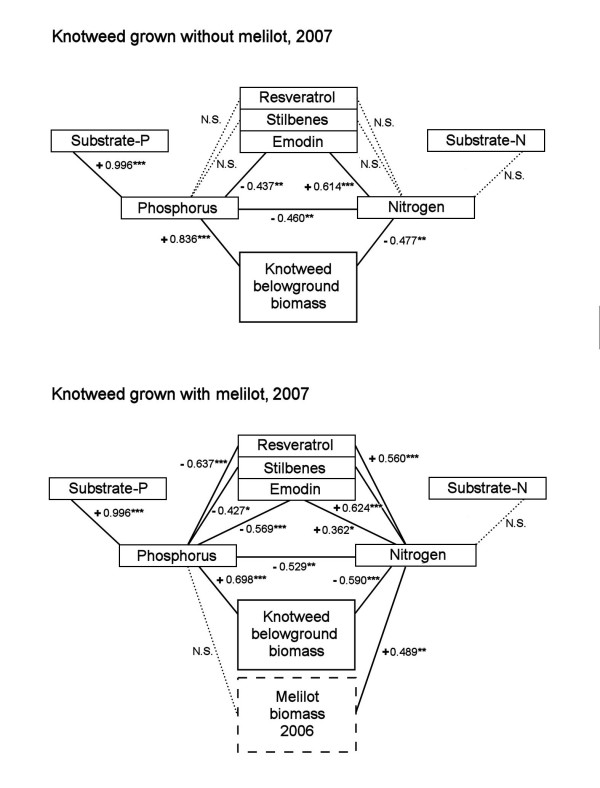
**Diagram of relationships between root and rhizome *Reynoutria *× *bohemica *characteristics (resveratrol, stilbenes, emodin, phosphorus and nitrogen contents and biomass) measured in 2007, melilot biomass measured in preceding year and phosphorus and nitrogen contents in the substrate, all in the absence and presence of melilot, respectively**. Correlation coefficients are shown in cases of relationships that were significant at P ≤ 0.05.

A synthesis of our data on plant biomass, resveratrol and its derivatives, emodin, nitrogen and phosphorus, and the relationships between these variables, are shown in Fig. 11.

Regardless of whether or not melilot was present, the biomass of roots and rhizomes was positively correlated with phosphorus content and negatively correlated with nitrogen content. Nitrogen content was negatively correlated with phosphorus content. The phosphorus content of the plants was highly positively correlated with the phosphorus content of the substrate. However, the total nitrogen content of the substrate was not correlated with the nitrogen content of knotweed rhizomes and roots (Fig. 11). In the absence of melilot, there were no relationships between either phosphorus or nitrogen and resveratrol or resveratrol derivatives. There was, however, a negative correlation between phosphorus and emodin and a positive correlation between nitrogen and emodin (Fig. 11).

The presence of melilot increased the concentration of resveratrol and/or resveratrol derivatives (Figs. 3b and 4b), but did not increase the concentration of phosphorus in knotweed grown on low-phosphorus substrates (Fig. 7). These resulted in a negative relationship between phosphorus and resveratrol and/or resveratrol derivatives. On the other hand, knotweed plants grown on a high-phosphorus substrate (compost) exhibited a high phosphorus content but low contents of resveratrol and/or resveratrol derivatives. The presence of melilot also revealed a positive relationship between nitrogen and resveratrol or resveratrol derivatives because it increased both nitrogen content and the content of resveratrol or resveratrol-derivatives (Figs. 3b, 4b, 6b and 11). Moreover, we observed a significant relationship between melilot biomass in 2006 and nitrogen content in the rhizomes and roots of knotweed in 2007 (Fig. 11). Also, there was a difference in knotweed root and rhizome nitrogen content between 2006 and 2007 that was correlated (r = 0.399**) with the quantity of melilot biomass produced in 2006. These results provide evidence that the nitrogen deposited in knotweed roots and rhizomes was supplied by melilot and its rhizobia.

A significant negative relationship was found between resveratrol and both nitrogen (r = -0.80*) and phosphorus (r = -0.95**) in grapevine leaves [31]. Also, vine berries with high nitrogen levels exhibited a decreased resveratrol content [5]. The negative relationship between resveratrol and phosphorus is in accordance with our findings. However, we found a positive relationship between resveratrol and nitrogen in the presence of melilot and no significant relationship in the absence of melilot. Nitrogen fixation of rhizobia has a high energy cost because the fixation of 1 gram of nitrogen requires 10 g glucose under favourable conditions http://www.biologie.uni-hamburg.de/b-online/e34/34b.htm. If glucose is transported from knotweed to melilot to cover the energy spent on nitrogen fixation, less glucose would be available to form resveratrol glucosides in a knotweed-melilot-rhizobia system that fixed relatively high amounts of nitrogen. Thus, relative to the quantity of resveratrol glucosides, more resveratrol would be observed. In our pot experiment, the ratio of resveratrol to resveratrol glucosides in knotweed was indeed significantly higher in the presence of melilot (0.10) than in the absence of melilot (0.03) for low-nutrient clay and loess.

**Figure 12:**
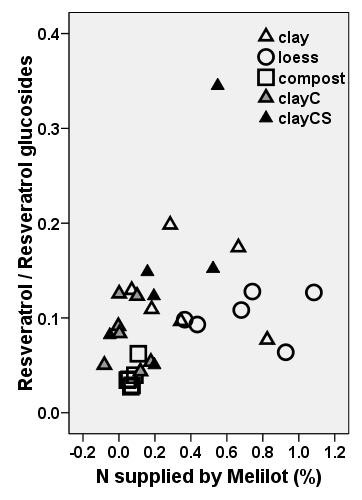
**Relationship between the ratio of resveratrol contained in its aglycons (resveratrol and piceatannol) to its glucosides (astringines, piceid, resveratrolosid), and differences in nitrogen concentration in belowground biomass of *Reynoutria *× *bohemica *grown with *Melilotus alba *(measured values) and without melilot (average per substrate)**. ClayC = clay enriched with slow-release biofertilizer Conavit; ClayCS = clay enriched with Conavit and arbuscular-mycorrhizal product Symbivit, both produced by Symbiom Ltd.

Not only the presence of melilot but also the efficiency of melilot to fix nitrogen (expressed as the difference in N concentration in the belowground biomass of knotweed between plants with and without melilot) was significantly correlated (r = 0.350*) with the ratio of resveratrol to resveratrol glucoside (Fig. 12). This clearly depicts the differences between all of the substrates. Compost is revealed to be a substrate with a low efficiency of N fixation and, at the same time, with a higher proportion of resveratrol glucosides compared with its aglycones. The opposite is true for the clayish low-nutrient substrates, clay and loess. Our data thus suggest the existence of glucose transport between the two plants, knotweed and melilot, and illustrate how costly nitrogen fixation is.

As for the transport of nitrogen, the following observations have been made: 1) the rhizobia bacteroid membrane is permeable to amino acids [32]; 2) bacteroids cycle amino acids to the host plant http://www.biologie.uni-hamburg.de/b-online/e34/34b.htm; 3) roots exude both amino acids and sugars [33]; and 4) fungal hyphae are able to transport nitrogen [34], even amino acids [35], and can transport sugars both passively and actively [36]. The plants in our system are clearly interconnected by fungal hyphae, as the melilot acts as a donor plant of mycorrhizal fungi; vesicules and hyphae, but no arbuscules, have been found in the roots of knotweed growing together with melilot, but none have been observed in the absence of melilot. Transport of substances via hyphae is to be expected in our system. However, we did not examine the mechanisms of transport, which require further study.

## Conclusions

A three year field experiment revealed that 2.6 t of dry mass and 8.5 kg of stilbenes are produced per hectare of knotweed. Spoil bank soils are thus promising areas to grow knotweed, namely this hexaploid clone of *R. *× *bohemica*, as a medicinal plant for production of resveratrol and resveratrol-containing substances.

In a pot experiment, the highest knotweed biomass production was observed in plants grown on high-nutrient substrates, namely compost. However, the concentrations of organic constituents studied were higher in plants grown in the presence of melilot on clayish low-nutrient substrates. Melilot significantly increased the contents of resveratrol-derivatives in knotweed roots and rhizomes in plants grown on clay, clayCS and loess. On most substrates, the contents of nitrogen and emodin in the roots and rhizomes of knotweed were also increased by the presence of melilot. Melilot showed a more pronounced effect than the substrate on production of resveratrol derivatives and emodin. Relationships were found between nitrogen, phosphorus, emodin, and belowground knotweed biomass. The presence of melilot revealed additional relationships between these characteristics, and resveratrol and resveratrol derivatives. Knotweed phosphorus was predominantly taken up from the substrate and the content of knotweed phosphorus was negatively correlated with resveratrol derivatives. On the other hand, knotweed nitrogen was primarily supplied by melilot and was found to be positively correlated with resveratrol derivatives.

The following generalised schemes for knotweed roots and rhizomes grown with melilot on low (1) and/or high (2) nutrient substrates can be thus formulated: (1) Low biomass ↔ Low phosphorus concentration in biomass ↔ High nitrogen concentration in biomass ↔ Limitation or co-limitation of plant production by phosphorus ↔ High resveratrol, resveratrol derivatives and emodin production; and/or (2) High biomass ↔ High phosphorus concentration in biomass ↔ Low nitrogen concentration in biomass ↔ Limitation of plant production by nitrogen ↔ Low resveratrol, resveratrol derivatives and emodin production.

The efficiency of nitrogen fixation (expressed as the difference in N concentration in knotweed belowground biomass between plants grown with and without melilot) was significantly correlated (r = 0.509**) with the ratio of resveratrol to resveratrol glucoside. This indicates that knotweed contributed to the energy cost of nitrogen fixation for melilot and that there is an exchange of organic substances between these two plant species. There appeared to be differences between the substrates. Compost was revealed to have a low efficiency of N fixation and, at the same time, showed a higher proportion of resveratrol glucosides compared with its aglycones. The opposite was true for the clayish low-nutrient substrates, clay and loess.

## Methods

### Pot experiment

#### Substrates

Clay of miocene origin was obtained from spoil banks that were made up of the same material as the soil in the field experiment (both with 50% of 10-50 μm particles), loess from nearby loess deposits and compost was that used for dump reclamation. The chemical composition of the substrates is shown in Table 2. Ten pots were filled with 7.25 kg (6 l) of clay (bottom) each and 2 l of one of the following substrates: loess (2.13 kg); "compost", composed of a 1:1 mixture of common compost and a cellulose-rich paper mill by-product called Lignocel (1.4 kg); or clay (2.4 kg) enriched with a slow-release biofertilizer Conavit^® ^("clayC"); or clay enriched with Conavit (30 g) and 50 ml (47 g) of arbuscular-mycorrhizal product Symbivit^® ^("clayCS"). For technical sheet and composition of both products see http://www.symbiom.cz. A mixture of six mycorrhizal fungi species (*Glomus etunicatum, G. microagregatum, G. intraradices, G. claroideum, G. mosseae *and *G. geosporum*) with at least 80,000 living propagules per litre in zeolit or spongilit was added to each pot, in addition to expanded clay enriched with natural fertilizer. Conavit is a completely natural slow nutrient releasing fertilizer composed of sea algae, humus substances, ground minerals and rocks, and is a natural source of keratin. A quantity of Conavit corresponding to 160 kg/ha was applied. Symbivit was added to the Conavit-treated pots on top of the bottom clay layer. The bottom layer of clay had a texture of larger lumps, while the overlying material was broken up into smaller particles. Twenty pots of each variant were prepared for a total of 100 pots. The pots were thoroughly wetted and kept in the greenhouse at 18-27°C. During the summer, the whole set was transferred outdoors to the experimental garden and was kept moist using automatic drop irrigation as necessary.

**Table 2:** Chemical composition of the substrates and fertilizers used in the experiment

Substrate	pH(H_2_O)	pH(KCl)	Conductivity	N	C	P	K	Ca	Mg	Na
			**μS**	**%**	**%**	**ppm**	**ppm**	**ppm**	**ppm**	**ppm**

Clay	7.26	7.12	718	0.08	5.60	20.4	693	2651	527	411

Loess	8.22	7.57	404	0.26	1.59	10.5	823	8172	1088	1506

Compost	6.97	6.92	395	2.18	17.58	652	7314	11118	2536	2296

Conavit	7.96	7.73	1354	2.45	9.16	65.5	18550	1536	640	3839

Symbivit	7.99	7.65	688	0.23	1.14	10.2	7483	360	158	2230

N, C - total (Carlo-Erba CHN analyzer); P (Olsen); K, Ca, Mg and Na - Mehlich II extracts.

#### Plants

At the start of the experiment, November 18, 2005, segments of *R. *× *bohemica *rhizomes (hexaploid, n = 66) that had been pre-cultivated in peat were carefully prepared. Each pot received a segment of washed rhizome with a known fresh weight and a known number of buds. The average fresh weight of a segment was 3.3 g and the average bud number was 1.6. The bud numbers did not differ significantly between the variants. Approximately 40 additional segments of these rhizomes were each inserted into a small pot of perlite in order to produce plantlets in case some of the plants in the experimental pots failed to grow. This proved to be a great advantage because some of the rhizomes, especially those from the variant grown with Conavit, did not produce any plantlets. This is probably due to the adverse effect of humic substances on the growth of fine roots. The dormant rhizomes were later exchanged for mature plantlets from the perlite pots. The pre-grown plantlets continued their growth without restriction, regardless of which type of substrate they were transplanted into.

After three months, the *R. *× *bohemica *plants were well established and white melilot seeds (*Melilotus albus *cv Krajová) were added to 10 out of the 20 pots of each variant. The ability of the seeds to germinate was assessed prior to seeding and was found to be 57% based on the average from 10 Petri dishes, each with 25 seeds. There are approximately 500 seeds in one gram.

After the first season, the plants were harvested in September 2006. We measured twig numbers, lengths and dry masses of both *Reynoutria *and *Mellilotus*, and excised 100 mm segments of the new rhizomes, which formed alongside the pot wall, for chemical analyses. The ramification of the branches was also taken into account; the lengths of all the main branches rising from the soil, as well as the lengths of all of the side branches, were measured and evaluated. Fine roots were sampled, while knotweed roots were hand-separated from the melilot roots, and both were stained and inspected for the presence of mycorrhiza. The experiment was terminated after the second season in September 2007. At the end of the experiment, both the aboveground and belowground biomass were measured, the fine roots were sampled for mycorrhiza and larger roots and rhizomes were thoroughly washed using air and water pressure. These were then dried and ground for analysis. Melilot was allowed to grow without restriction during the first season, but plants were repeatedly cut during the second season to maintain a height of 30 cm.

### Field experiment

The centre of the 1 ha experimental non-irrigated field is at a location of 50°35'N, 13°52'E. This experiment field is a former spoil bank that was transformed into an arable field by organic manuring and ploughing and still shows a high clay content. In April 2006, 15-20 cm long rhizomes of pre-cultivated *R. *× *bohemica *(n = 66) were planted with a spacing of 100 × 70 cm and were immediately covered with soil. Ten plants were randomly sampled on each sampling day in July and September of 2006, and in May, July and September of 2007 and 2008. Plants were then washed and dried (at 60°C) aboveground and the belowground biomass was measured. Six samples from each set were analysed for the same stilbenes and emodin as the samples from the pot experiment.

#### Organic analyses

**Figure 13:**
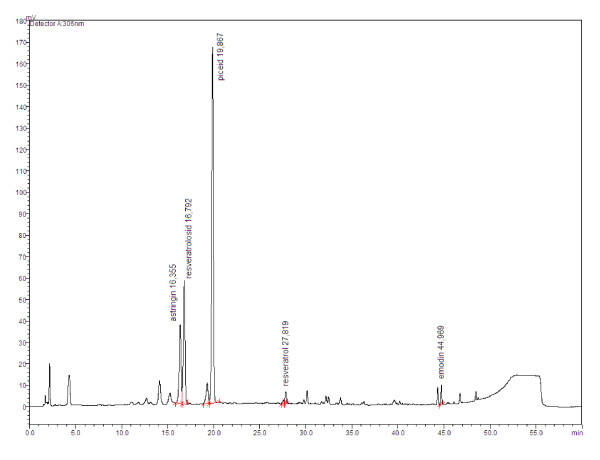
**A typical HPLC record of the stilbenes and emodin measured in knotweed rhizomes**.

The stilbenes - resveratrol, piceatannol and its glycosides (piceid, resveratrolosid, astringines), were analysed along with emodin in samples of knotweed rhizomes and roots. Dry and finely ground samples (0.01 mm sieve) were extracted with 60% ethanol, and the extracts were analysed using HPLC (Shimadzu LC2010C HT using Phenomenex Synergi Hydro-RP 80A, 250 × 4,6 mm, 4 μm 30°C, flow rate 1.5 ml/min, detection at 306 nm using a mobile phase gradient from 7% to 90% B; A: 10 mM acetate ammonium at pH 4.15; B: ACN g.g). Fig. 13 shows a typical record of the stilbenes and emodin measured by this method.

#### Assessment of mycorrhiza

A modification of a common mycological staining procedure was used to clear and stain samples. The soil samples were rinsed with water on a sieve. The roots were hand-separated, cut into 1-2 cm segments, washed with 10% (w/v) KOH solution and stained with 0.05% (w/v) trypan blue in lactoglycerol. Root segments were viewed under a microscope (Olympus BX41) at ×100 or ×200 magnification and were screened for mycorrhizal colonisation. The presence or absence of AM colonisation (arbuscules, vesicles and internal hyphae) was determined. The degree of mycorrhizal colonisation was evaluated using the grid-line intersect method at ×50 magnification under a dissecting microscope. The frequency (F) and intensity (M) of mycorrhizal colonisation were also calculated [37].

#### Data analysis

The data were analysed using SPSS 15.0 (SPSS, Cary, NC, USA) statistical software. Normality of the data was tested and non-normally distributed data were transformed by rank. A two- or three-way ANOVA was used to test the differences between the variants, while a Tukey's test was applied to compare the individual means. A Pearson's correlation was calculated to evaluate relationships between the growth characteristics measured. If not otherwise indicated, the significance level was set at P ≤ 0.05 and is indicated by a single asterisk. Two asterisks indicate a significance level of P ≤ 0.01, while three asterisks indicate a significance level of P ≤ 0.001.

## Authors' contributions

MK conceived the study, coordinated the experiments and drafted the manuscript. TF performed the statistical analyses, prepared graphs and commented on the draft text. KB performed the mycorrhizal part of the study. HK participated substantially in the coordination of the experimental work and group members. KP adjusted and performed the organic chemical analyses. MV designed the experiment and contributed to the written manuscript. All authors read and approved the final paper.

## References

[B1] Mandak B Bimova K Pysek P Stepanek J Plackova I Isoenzyme diversity in Reynoutria (Polygonaceae) taxa: escape from sterility by hybridization Plant Systematics and Evolution 2005 253 219 230 10.1007/s00606-005-0316-6

[B2] Mandak B Pysek P Lysak M Suda J Krahulcova A Bimova K Variation in DNA-ploidy levels of Reynoutria taxa in the Czech Republic Annals of Botany 2003 92 265 272 10.1093/aob/mcg141 12876190 PMC4243663

[B3] Pysek P Brock JH Bimova K Mandak B Jarosik V Koukolikova I Pergl J Stepanek J Vegetative regeneration in invasive Reynoutria (Polygonaceae) taxa: The determinant of invasibility at the genotype level American Journal of Botany 2003 90 1487 1495 10.3732/ajb.90.10.1487 21659101

[B4] Chrtek J Chrtkova A *Reynoutria *× *bohemica *nový køíženec z èeledi rdesnovitých. (*Reynoutria *× *bohemica*, a new hybrid of Polygonaceae) Èas Nár Muz, Ser Nat 1983 152 120

[B5] Bavaresco L Pezzutto S Ragga A Ferrari F Trevisan M Effect of nitrogen supply on trans-resveratrol concentration in berries of Vitis vinifera L. cv. Cabernet Sauvignon Vitis 2001 40 229 230

[B6] Schubert R Fischer R Hain R Schreier PH Bahnweg G Ernst D Sandermann H An ozone-responsive region of the grapevine resveratrol synthase promoter differs from the basal pathogen-responsive sequence Plant Molecular Biology 1997 34 417 426 10.1023/A:1005830714852 9225853

[B7] Soleas GJ Diamandis EP Goldberg DM Resveratrol: a molecule whose time has come? And gone? Clin Biochem 1997 30 91 113 10.1016/S0009-9120(96)00155-5 9127691

[B8] Ferry-Dumazet H Garnier O Mamani-Matsuda M Vercauteren J Belloc F Billiard C Dupouy M Thiolat D Kolb JP Marit G Reiffers J Mossalayi MD Resveratrol inhibits the growth and induces the apoptosis of both normal and leukemic hematopoietic cells Carcinogenesis 2002 23 1327 1333 10.1093/carcin/23.8.1327 12151351

[B9] Roman V Billard C Kern C Ferry-Dumazet H Izard JC Mohammad R Mossalayi DM Kolb JP Analysis of resveratrol-induced apoptosis in human B-cell chronic leukaemia British Journal of Haematology 2002 117 842 851 10.1046/j.1365-2141.2002.03520.x 12060119

[B10] Ulrich S Wolter F Stein JM Molecular mechanisms of the chemopreventive effects of resveratrol and its analogs in carcinogenesis Molecular Nutrition & Food Research 2005 49 452 461 10.1002/mnfr.200400081 15830333

[B11] Wolter F Ulrich S Stein J Molecular mechanisms of the chemopreventive effects of resveratrol and its analogs in colorectal cancer: Key role of polyamines? Journal of Nutrition 2004 134 3219 3222 15570015 10.1093/jn/134.12.3219

[B12] El-Mowafy AM Alkhalaf M Resveratrol activates adenylyl-cyclase in human breast cancer cells: a novel, estrogen receptor-independent cytostatic mechanism Carcinogenesis 2003 24 869 873 10.1093/carcin/bgg015 12771030

[B13] Carando S Teissedre PL Waffo-Teguo P Cabanis JC Deffieux G Merillon JM High-performance liquid chromatography coupled with fluorescence detection for the determination of trans-astringin in wine Journal of Chromatography A 1999 849 617 620 10.1016/S0021-9673(99)00595-6 10457456

[B14] Merillon JM Fauconneau B Teguo PW Barrier L Vercauteren J Huguet F Antioxidant activity of the stilbene astringin, newly extracted from Vitis vinifera cell cultures Clinical Chemistry 1997 43 1092 1093 9191572

[B15] Vitrac X Bornet A Vanderlinde R Valls J Richard T Delaunay JC Merillon JM Teissedre PL Determination of stilbenes (delta-viniferin, trans-astringin, trans-piceid, cis- and trans-resveratrol, epsilon-viniferin) in Brazilian wines Journal of Agricultural and Food Chemistry 2005 53 5664 5669 10.1021/jf050122g 15998130

[B16] de Lima MTR Waffo-Teguo P Teissedre PL Pujolas A Vercauteren J Cabanis JC Merillon JM Determination of stilbenes (trans-astringin, cis- and trans-piceid, and cis- and trans-resveratrol) in Portuguese wines Journal of Agricultural and Food Chemistry 1999 47 2666 2670 10.1021/jf9900884 10552542

[B17] Blumenstein I Keseru B Wolter F Stein J The chemopreventive agent resveratrol stimulates cyclic AMP - Dependent chloride secretion in vitro Clinical Cancer Research 2005 11 5651 5656 10.1158/1078-0432.CCR-04-2674 16061885

[B18] Fremont L Gozzelino MT Linard A Response of plasma lipids to dietary cholesterol and wine polyphenols in rats fed polyunsaturated fat diets Lipids 2000 35 991 999 10.1007/s11745-000-0610-2 11026620

[B19] Ibern-Gómez M Roig-Pérez S de la Torre-Boronat MC Resveratrol and piceid levels in natural and blended peanut butters Journal of Agricultural and Food Chemistry 2009 48 6352 6354 10.1021/jf000786k 11312807

[B20] Utrup LJ Norris JH Nodulin gene expression in effective root nodules of white sweetclover (Melilotus alba Desr) and in ineffective nodules elicited by mutant strains of Rhizobium meliloti Journal of Experimental Botany 1996 47 195 202 10.1093/jxb/47.2.195

[B21] Zahran HH Rhizobia from wild legumes: diversity, taxonomy, ecology, nitrogen fixation and biotechnology Journal of Biotechnology 2001 91 143 153 10.1016/S0168-1656(01)00342-X 11566386

[B22] Harley JL Harley EL A Checklist of Mycorrhiza in the British Flora - Addenda, Errata and Index New Phytologist 1987 107 741 749 10.1111/j.1469-8137.1987.tb00912.x

[B23] Fuiyoshi M Masuzawa T Kagawa A Nakatsubo T Successional changes in mycorrhizal type in the pioneer plant communities of a subalpine volcanic desert on Mt. Fuji, Japan Polar Biosci 2005 18 60 72

[B24] Ocampo JA Martin J Hayman DS Influence of Plant Interactions on Vesicular-Arbuscular Mycorrhizal Infections. 1. Host and Non-Host Plants Grown Together New Phytologist 1980 84 27 10.1111/j.1469-8137.1980.tb00746.x

[B25] Sykorova Z Rydlova J Vosatka M Establishment of mycorrhizal symbiosis in Gentiana verna Folia Geobotanica 2003 38 177 189 10.1007/BF02803150

[B26] Brock JH Standing crop of Reynoutria japonica in the autumn of 1991 in the United Kingdom Preslia 1995 66 37 43

[B27] Craine JM Morrow C Stock WD Nutrient concentration ratios and co-limitation in South African grasslands New Phytologist 2008 179 829 836 10.1111/j.1469-8137.2008.02513.x 18537887

[B28] Craine JM Resource strategies of wild plants. Princeton 2009

[B29] Koerselman W Meuleman AFM The vegetation N:P ratio: a new tool to detect the nature of nutrient limitation Journal of Applied Ecology 1996 33 1441 1450 10.2307/2404783

[B30] Gusewell S N:P ratios in terrestrial plants: variation and functional significance New Phytologist 2004 164 243 266 10.1111/j.1469-8137.2004.01192.x 33873556

[B31] Bavaresco L Fregoni M Petegolli D Effect of Nitrogen and Potassium Fertilizer on Induced Resveratrol Synthesis in 2 Grapevine Genotypes Vitis 1994 33 175 176

[B32] Lodwig EM Hosie AHF Bourdes A Findlay K Allaway D Karunakaran R Downie JA Poole PS Amino-acid cycling drives nitrogen fixation in the legume-*Rhizobium *symbiosis Nature 2003 422 722 726 10.1038/nature01527 12700763

[B33] Naher UA Radziah O Halimi MS Shamsuddin ZH Mohd Razi I Effect of inoculation of inoculation on root exudates carbon sugar and amino acids production of different rice varieties Research Journal of Microbiology 2008 3 580 587 10.3923/jm.2008.580.587

[B34] Leigh J Hodge A Fitter AH Arbuscular mycorrhizal fungi can transfer substantial amounts of nitrogen to their host plant from organic material New Phytologist 2009 181 199 207 10.1111/j.1469-8137.2008.02630.x 18811615

[B35] Guether M Neuhauser B Balestrini R Dynowski M Ludewig U Bonfante P A mycorrhizal-specific ammonium transporter from Lotus japonicus acquires nitrogen released by arbuscular mycorrhizal fungi Plant Physiology 2009 150 73 83 10.1104/pp.109.136390 19329566 PMC2675747

[B36] Bago B Pfeffer PE Shachar-Hill Y Carbon Metabolism and Transport in Arbuscular Mycorrhizas Plant Physiology 2000 124 949 958 10.1104/pp.124.3.949 11080273 PMC1539291

[B37] Trouvelot A Kough JL Gianinazzi-Pearson V, Gianinazzi S Mesure du taux de mycorhization VA d'un systeme radiculaire. Recherche de méthodes d'estimation ayant une signification fonctionelle Physiological and Genetical Aspects of Mycorrhizae 1986 Paris: INRA Press 217 221

